# Supplementary material for: Time and Mode of Epidemic HCV-2 Subtypes Spreading in Europe: Phylodynamics in Italy and Albania
Source: Diagnostics (Basel). 2021 Feb 17;11(2):327. doi: 10.3390/diagnostics11020327 (PMC7922790; doi:10.3390/diagnostics11020327)
Supplement: Supplementary file 1 [file diagnostics-11-00327-s001.zip › Supplementary files/Supplementary Table S1 .docx]

| **Rates** | **Location 1** | **Location 2** | **Bayes Factor** |
| --- | --- | --- | --- |
| 1 | IT | VE | 86827.07 |
| 2 | IT | NL | 86827.07 |
| 3 | ID | SR | 86827.07 |
| 4 | AR | IT | 86827.07 |
| 5 | IT | TN | 14463.14 |
| 6 | CN | ID | 7226.74 |
| 7 | ID | NL | 5417.65 |
| 8 | AL | IT | 4560.7 |
| 9 | FR | IT | 3765.86 |
| 10 | CM | FR | 2108.32 |
| 11 | BF | GH | 161.63 |
| 12 | FR | GW | 96.64 |
| 13 | RU | US | 73.69 |
| 14 | GH | NE | 47.67 |
| 15 | GH | IT | 29.4 |
| 16 | GH | SP | 22.94 |
| 17 | GH | SR | 22.35 |
| 18 | GH | MA | 21.54 |
| 19 | FR | VN | 13.71 |
| 20 | EE | RU | 13.01 |
| 21 | FR | GH | 12.21 |
| 22 | GH | GW | 11.74 |
| 23 | IT | RU | 9.94 |
| 24 | ID | RU | 7.14 |
| 25 | AL | CA | 6.48 |
| 26 | NL | RU | 6.38 |
| 27 | CA | NL | 5.77 |
| 28 | EE | IT | 5.65 |
| 29 | CM | VN | 5.22 |
| 30 | CA | ID | 4.25 |
| 31 | NE | SR | 4.15 |
| 32 | CA | IT | 3.68 |
| 33 | ID | VN | 3.43 |
