# Supplementary material for: Time and Mode of Epidemic HCV-2 Subtypes Spreading in Europe: Phylodynamics in Italy and Albania
Source: Diagnostics (Basel). 2021 Feb 17;11(2):327. doi: 10.3390/diagnostics11020327 (PMC7922790; doi:10.3390/diagnostics11020327)
Supplement: Supplementary file 1 [file diagnostics-11-00327-s001.zip › Supplementary files/Supplementary Table S2.docx]

| Accession number | Sample Location | Sample date | Age | Sex | Sample Code | HCV-2 Subtype |
| --- | --- | --- | --- | --- | --- | --- |
| AF037248 | Burkina Faso | 1995 |  |  | 1BF@95 | 2j |
| AF037249 | Burkina Faso | 1995 |  |  | 2BF@95 | 2j |
| AF037250 | Burkina Faso | 1995 |  |  | 3BF@95 | 2b |
| AF037251 | Burkina Faso | 1995 |  |  | 4BF@95 | 2j |
| AF037252 | Burkina Faso | 1995 |  |  | 5BF@95 | 2a |
| AF388506 | Russia | 1999 |  |  | 6RU@99 | 2c |
| AF515980 | France | 1999 |  |  | 7FR@99 | 2c |
| AF515981 | France | 1999 |  |  | 8FR@99 | 2c |
| AF515995 | France | 2000 |  |  | 9FR@00 | 2c |
| AF515996 | France | 2000 |  |  | 10FR@00 | 2c |
| AY236367 | Ghana | 2000 |  |  | 11GH@00 | 2a |
| AY236368 | Ghana | 2000 |  |  | 12GH@00 | 2j |
| AY236371 | Ghana | 2000 |  |  | 13GH@00 | 2j |
| AY236372 | Ghana | 2000 |  |  | 14GH@00 | 2c |
| AY236373 | Ghana | 2000 |  |  | 15GH@00 | 2c |
| AY236374 | Ghana | 2000 |  |  | 16GH@00 | 2a |
| AY236375 | Ghana | 2000 |  |  | 17GH@00 | 2a |
| AY236376 | Ghana | 2000 |  |  | 18GH@00 | 2m |
| AY236377 | Ghana | 2000 |  |  | 19GH@00 | 2c |
| AY236378 | Ghana | 2000 |  |  | 20GH@00 | 2a |
| AY236379 | Ghana | 2000 |  |  | 21GH@00 | 2c |
| AY236380 | Ghana | 2000 |  |  | 22GH@00 | 2a |
| AY236382 | Ghana | 2000 |  |  | 23GH@00 | 2j |
| AY236383 | Ghana | 2000 |  |  | 24GH@00 | 2j |
| AY236384 | Ghana | 2000 |  |  | 25GH@00 | 2a |
| AY236385 | Ghana | 2000 |  |  | 26GH@00 | 2j |
| AY236386 | Ghana | 2000 |  |  | 27GH@00 | 2a |
| AY236387 | Ghana | 2000 |  |  | 28GH@00 | 2c |
| AY257076 | Cameroon | 1998 |  |  | 29CM@98 | 2j |
| AY257079 | Cameroon | 1998 |  |  | 30CM@98 | 2a |
| AY257080 | Cameroon | 1998 |  |  | 31CM@98 | 2j |
| AY257081 | Cameroon | 1998 |  |  | 32CM@98 | 2j |
| AY257088 | Cameroon | 1998 |  |  | 33CM@98 | 2j |
| AY257099 | Cameroon | 1998 |  |  | 34CM@98 | 2a |
| AY257100 | Cameroon | 1998 |  |  | 35CM@98 | 2a |
| AY944638 | Italy | 1996 |  |  | 36IT@96 | 2c |
| AY944639 | Italy | 1996 |  |  | 37IT@96 | 2c |
| AY944640 | Italy | 1996 |  |  | 38IT@96 | 2c |
| AY944662 | Italy | 1996 |  |  | 39IT@96 | 2c |
| AY944678 | Italy | 1996 |  |  | 40IT@96 | 2c |
| AY944682 | Italy | 1996 |  |  | 41IT@96 | 2c |
| DQ220854 | France | 2002 |  |  | 42FR@02 | 2c |
| EF195025 | Estonia | 1999 |  |  | 43EE@99 | 2c |
| EF195026 | Estonia | 2000 |  |  | 44EE@00 | 2c |
| EF195027 | Estonia | 2004 |  |  | 45EE@04 | 2c |
| GQ153896 | Guinea Bissau | 2005 |  |  | 46GW@05 | 2b |
| GQ153897 | Guinea Bissau | 2005 |  |  | 47GW@05 | 2b |
| GQ153898 | Guinea Bissau | 2005 |  |  | 48GW@05 | 2b |
| GQ153899 | Guinea Bissau | 2005 |  |  | 49GW@05 | 2j |
| GQ153900 | Guinea Bissau | 2005 |  |  | 50GW@05 | 2q |
| GQ153901 | Guinea Bissau | 2005 |  |  | 51GW@05 | 2j |
| GQ153902 | Guinea Bissau | 2005 |  |  | 52GW@05 | 2b |
| GQ153903 | Guinea Bissau | 2005 |  |  | 53GW@05 | 2j |
| GQ153904 | Guinea Bissau | 2005 |  |  | 54GW@05 | 2q |
| GQ153905 | Guinea Bissau | 2005 |  |  | 55GW@05 | 2a |
| GQ153906 | Guinea Bissau | 2005 |  |  | 56GW@05 | 2b |
| GQ153907 | Guinea Bissau | 2005 |  |  | 57GW@05 | 2j |
| GQ153908 | Guinea Bissau | 2005 |  |  | 58GW@05 | 2a |
| GQ153909 | Guinea Bissau | 2005 |  |  | 59GW@05 | 2a |
| GQ153911 | Guinea Bissau | 2005 |  |  | 61GW@05 | 2j |
| GQ418359 | Indonesia | 2007 |  |  | 62ID@07 | 2a |
| GQ418361 | Indonesia | 2006 |  |  | 63ID@06 | 2a |
| GQ418362 | Indonesia | 2006 |  |  | 64ID@06 | 2a |
| GQ418363 | Indonesia | 2006 |  |  | 65ID@06 | 2a |
| GQ387950 | Russia | 2001 |  |  | 66RU@01 | 2a |
| GQ387951 | Russia | 2002 |  |  | 67RU@02 | 2a |
| GQ387952 | Russia | 2002 |  |  | 68RU@02 | 2c |
| GQ387953 | Russia | 2001 |  |  | 69RU@01 | 2c |
| GQ387996 | Russia | 2002 |  |  | 70RU@02 | 2a |
| GQ387997 | Russia | 2002 |  |  | 71RU@02 | 2a |
| GQ387998 | Russia | 2002 |  |  | 72RU@02 | 2a |
| GQ387999 | Russia | 2003 |  |  | 73RU@03 | 2a |
| GQ388000 | Russia | 2002 |  |  | 74RU@02 | 2a |
| GQ388001 | Russia | 2002 |  |  | 75RU@02 | 2c |
| GU054420 | France | 1991 |  |  | 76FR@91 | 2j |
| GU054429 | France | 2001 |  |  | 77FR@01 | 2c |
| GU054434 | France | 2001 |  |  | 78FR@01 | 2b |
| GU054435 | France | 2002 |  |  | 79FR@02 | 2j |
| GU054438 | France | 2002 |  |  | 80FR@02 | 2j |
| GU054439 | France | 2002 |  |  | 81FR@02 | 2b |
| GU054440 | France | 2002 |  |  | 82FR@02 | 2j |
| GU054444 | France | 2002 |  |  | 83FR@02 | 2j |
| GU054445 | France | 2003 |  |  | 84FR@03 | 2c |
| GU054451 | France | 2005 |  |  | 86FR@05 | 2j |
| AB523261 | Vietnam | 2007 |  |  | 87VN@07 | 2j |
| GU441419 | Indonesia | 2008 |  |  | 88ID@08 | 2a |
| GU441420 | Indonesia | 2008 |  |  | 89ID@08 | 2a |
| GU441443 | Indonesia | 2008 |  |  | 90ID@08 | 2a |
| GU441445 | Indonesia | 2007 |  |  | 91ID@07 | 2a |
| GU441446 | Indonesia | 2008 |  |  | 92ID@08 | 2a |
| GU441456 | Indonesia | 2008 |  |  | 93ID@08 | 2a |
| HM777358 | Venezuela | 2006 |  |  | 94VE@06 | 2j |
| HM777359 | Venezuela | 2005 |  |  | 95VE@05 | 2j |
| FN666429 | Spain | 2001 |  |  | 96SP@01 | 2q |
| FN666428 | Spain | 2002 |  |  | 97SP@02 | 2q |
| HM777346 | Venezuela | 1996 |  |  | 98VE@96 | 2c |
| HM777347 | Venezuela | 1996 |  |  | 99VE@96 | 2c |
| HM777348 | Venezuela | 2003 |  |  | 100VE@03 | 2c |
| HM777349 | Venezuela | 2004 |  |  | 101VE@04 | 2c |
| HM777350 | Venezuela | 2005 |  |  | 102VE@05 | 2c |
| HM777351 | Venezuela | 2005 |  |  | 103VE@05 | 2c |
| HM777352 | Venezuela | 2005 |  |  | 104VE@05 | 2c |
| HM777353 | Venezuela | 2005 |  |  | 105VE@05 | 2c |
| HM777354 | Venezuela | 2006 |  |  | 106VE@06 | 2c |
| HM777355 | Venezuela | 2006 |  |  | 107VE@06 | 2c |
| HM777356 | Venezuela | 2006 |  |  | 108VE@06 | 2c |
| HM777357 | Venezuela | 2007 |  |  | 109VE@07 | 2c |
| JF511066 | Argentine | 2000 |  |  | 110AR@00 | 2c |
| JF511072 | Argentine | 2001 |  |  | 111AR@01 | 2c |
| JF511073 | Argentine | 2001 |  |  | 112AR@01 | 2c |
| JF511077 | Argentine | 2008 |  |  | 113AR@08 | 2c |
| JF511078 | Argentine | 2008 |  |  | 114AR@08 | 2c |
| JF511079 | Argentine | 2005 |  |  | 115AR@05 | 2c |
| JF511080 | Argentine | 1999 |  |  | 116AR@99 | 2c |
| JF511081 | Argentine | 2003 |  |  | 117AR@03 | 2c |
| JF511082 | Argentine | 2003 |  |  | 118AR@03 | 2c |
| JF511120 | Argentine | 2004 |  |  | 119AR@04 | 2c |
| JF511121 | Argentine | 2004 |  |  | 120AR@04 | 2c |
| JF511122 | Argentine | 2004 |  |  | 121AR@04 | 2c |
| JF511123 | Argentine | 2004 |  |  | 122AR@04 | 2c |
| JF511124 | Argentine | 2004 |  |  | 123AR@04 | 2c |
| JF511125 | Argentine | 2004 |  |  | 124AR@04 | 2c |
| JF511126 | Argentine | 2004 |  |  | 125AR@04 | 2c |
| JF511127 | Argentine | 2004 |  |  | 126AR@04 | 2c |
| JF511128 | Argentine | 2004 |  |  | 127AR@04 | 2c |
| JF511129 | Argentine | 2004 |  |  | 128AR@04 | 2c |
| JF511130 | Argentine | 2007 |  |  | 129AR@07 | 2c |
| JF511131 | Argentine | 2000 |  |  | 130AR@00 | 2c |
| JF511132 | Argentine | 2005 |  |  | 131AR@05 | 2c |
| JF511133 | Argentine | 2005 |  |  | 132AR@05 | 2c |
| JF511134 | Argentine | 2005 |  |  | 133AR@05 | 2c |
| JF511135 | Argentine | 2006 |  |  | 134AR@06 | 2c |
| JF511136 | Argentine | 2007 |  |  | 135AR@07 | 2c |
| JF824343 | Canada | 2007 |  |  | 136CA@07 | 2a |
| JF824370 | Canada | 2007 |  |  | 137CA@07 | 2a |
| JF824423 | Canada | 2007 |  |  | 138CA@07 | 2a |
| JF722506 | Holland | 2001 |  |  | 139NL@01 | 2c |
| JF722517 | Holland | 2003 |  |  | 140NL@03 | 2c |
| JF722531 | Holland | 2006 |  |  | 141NL@06 | 2c |
| JF722550 | Holland | 2001 |  |  | 142NL@01 | 2c |
| JF722558 | Holland | 2001 |  |  | 143NL@01 | 2c |
| JF722580 | Holland | 2007 |  |  | 144NL@07 | 2c |
| JF722589 | Holland | 2003 |  |  | 145NL@03 | 2c |
| JQ679028 | Nigeria | 2007 |  |  | 146NE@07 | 2j |
| JQ679030 | Nigeria | 2007 |  |  | 148NE@07 | 2a |
| JQ679031 | Nigeria | 2007 |  |  | 149NE@07 | 2a |
| JQ679032 | Nigeria | 2007 |  |  | 150NE@07 | 2a |
| JQ679033 | Nigeria | 2007 |  |  | 151NE@07 | 2a |
| JQ679034 | Nigeria | 2007 |  |  | 152NE@07 | 2j |
| JQ679035 | Nigeria | 2007 |  |  | 153NE@07 | 2a |
| JQ679036 | Nigeria | 2007 |  |  | 154NE@07 | 2j |
| JQ746507 | Holland | 2002 |  |  | 155NL@02 | 2c |
| JX103080 | Vietnam | 2009 |  |  | 156VN@09 | 2a |
| KC197227 | France | 2008 |  |  | 157FR@08 | 2c |
| KC197228 | France | 2004 |  |  | 158FR@04 | 2c |
| KJ206319 | Tunisia | 2003 |  |  | 159TN@03 | 2c |
| KJ206320 | Tunisia | 2007 |  |  | 160TN@07 | 2c |
| KJ206321 | Tunisia | 2004 |  |  | 161TN@04 | 2c |
| KJ206322 | Tunisia | 2008 |  |  | 162TN@08 | 2c |
| KJ206323 | Tunisia | 2003 |  |  | 163TN@03 | 2c |
| KJ206324 | Tunisia | 2004 |  |  | 164TN@04 | 2c |
| KJ206325 | Tunisia | 2005 |  |  | 165TN@05 | 2c |
| KJ206326 | Tunisia | 2006 |  |  | 166TN@06 | 2c |
| KJ206327 | Tunisia | 2007 |  |  | 167TN@07 | 2c |
| KJ206328 | Tunisia | 2008 |  |  | 168TN@08 | 2c |
| KJ206329 | Tunisia | 2008 |  |  | 169TN@08 | 2c |
| KJ206330 | Tunisia | 2008 |  |  | 170TN@08 | 2c |
| KJ206331 | Tunisia | 2010 |  |  | 171TN@10 | 2c |
| KJ206332 | Tunisia | 2007 |  |  | 172TN@07 | 2c |
| KJ206333 | Tunisia | 2007 |  |  | 173TN@07 | 2c |
| KF793377 | China | 2013 |  |  | 174CN@13 | 2a |
| KF793384 | China | 2013 |  |  | 175CN@13 | 2a |
| KF793385 | China | 2013 |  |  | 176CN@13 | 2a |
| KF793394 | China | 2013 |  |  | 177CN@13 | 2a |
| KF793439 | China | 2013 |  |  | 178CN@13 | 2a |
| KF793457 | China | 2013 |  |  | 179CN@13 | 2a |
| KF793479 | China | 2013 |  |  | 180CN@13 | 2a |
| KF793486 | China | 2013 |  |  | 181CN@13 | 2a |
| KF793495 | China | 2013 |  |  | 182CN@13 | 2a |
| KF793497 | China | 2013 |  |  | 183CN@13 | 2a |
| KM587616 | USA | 2012 |  |  | 184US@12 | 2a |
| KM587620 | USA | 2012 |  |  | 185US@12 | 2c |
|  | Albania | 2014 |  | M | 196AL@14 | 2c |
|  | Albania | 2014 |  | M | 219AL@14 | 2c |
|  | Albania | 2015 |  | M | 220AL@15 | 2c |
|  | Albania | 2015 |  | M | 221AL@15 | 2c |
|  | Albania | 2007 |  | M | 201AL@07 | 2c |
|  | Albania | 2010 |  | F | 222AL@10 | 2c |
|  | Albania | 2007 |  | F | 202AL@07 | 2c |
|  | Albania | 2007 |  | F | 203AL@07 | 2c |
|  | Albania | 2011 |  | M | 187AL@11 | 2c |
|  | Albania | 2007 |  | M | 199AL@07 | 2c |
|  | Albania | 2007 |  | F | 204AL@07 | 2c |
|  | Albania | 2007 |  | F | 205AL@07 | 2c |
|  | Albania | 2007 |  | M | 206AL@07 | 2c |
|  | Albania | 2008 |  | F | 207AL@08 | 2c |
|  | Albania | 2008 |  | F | 208AL@08 | 2c |
|  | Albania | 2008 |  | F | 209AL@08 | 2c |
|  | Albania | 2011 |  | M | 188AL@11 | 2c |
|  | Albania | 2008 |  | F | 210AL@08 | 2c |
|  | Albania | 2009 |  | F | 190AL@09 | 2c |
|  | Albania | 2009 |  | M | 191AL@09 | 2c |
|  | Albania | 2009 |  | F | 211AL@09 | 2c |
|  | Albania | 2010 |  | M | 212AL@10 | 2c |
|  | Albania | 2014 |  | M | 192AL@14 | 2c |
|  | Albania | 2014 |  | F | 193AL@14 | 2c |
|  | Albania | 2014 |  | M | 197AL@14 | 2a |
|  | Albania | 2012 |  | M | 213AL@12 | 2c |
|  | Albania | 2011 |  | F | 214AL@11 | 2c |
|  | Albania | 2012 |  | F | 215AL@12 | 2c |
|  | Albania | 2013 |  | F | 189AL@13 | 2c |
|  | Albania | 2012 |  | F | 216AL@12 | 2c |
|  | Albania | 2012 |  | M | 217AL@12 | 2c |
|  | Albania | 2011 |  | M | 186AL@11 | 2c |
|  | Albania | 2012 |  | F | 218AL@12 | 2c |
|  | Albania | 2014 |  | M | 194AL@14 | 2c |
|  | Albania | 2007 |  | M | 198AL@07 | 2c |
|  | Albania | 2014 |  | M | 195AL@14 | 2c |
|  | Albania | 2007 |  | M | 200AL@07 | 2c |
|  | Italy | 2015 | 19 | F | M223IT@15 | 2c |
|  | Italy | 2015 | 71 | F | M224IT@15 | 2c |
|  | Italy | 2015 | 65 | M | M225IT@15 | 2c |
|  | Italy | 2015 | 80 | F | M226IT@15 | 2c |
|  | Italy | 2015 | 79 | F | M227IT@15 | 2c |
|  | Italy | 2015 | 58 | M | M228IT@15 | 2a |
|  | Italy | 2015 | 64 | F | M229IT@15 | 2c |
|  | Italy | 2015 | 41 | F | M230IT@15 | 2c |
|  | Italy | 2015 | 79 | M | M231IT@15 | 2c |
|  | Italy | 2015 | 71 | M | M232IT@15 | 2c |
|  | Italy | 2015 | 64 | M | M233IT@15 | 2b |
|  | Italy | 2015 | 82 | F | M234IT@15 | 2c |
|  | Italy | 2015 | 84 | M | M235IT@15 | 2c |
|  | Italy | 2015 | 81 | F | M236IT@15 | 2c |
|  | Italy | 2015 | 50 | F | M237IT@15 | 2b |
|  | Italy | 2015 | 82 | F | M238IT@15 | 2c |
|  | Italy | 2016 | 68 | F | M239IT@16 | 2c |
|  | Italy | 2016 | 85 | M | M240IT@16 | 2c |
|  | Italy | 2016 | 86 | F | M241IT@16 | 2c |
|  | Italy | 2016 | 48 | F | M242IT@16 | 2c |
|  | Italy | 2016 | 76 | F | M243IT@16 | 2c |
|  | Italy | 2016 | 78 | M | M244IT@16 | 2c |
|  | Italy | 2016 | 52 | F | M245IT@16 | 2c |
|  | Italy | 2016 | 84 | F | M246IT@16 | 2c |
|  | Italy | 2016 | 79 | M | M247IT@16 | 2c |
|  | Italy | 2016 | 50 | M | M248IT@16 | 2b |
|  | Italy | 2016 | 74 | M | M249IT@16 | 2c |
|  | Italy | 2016 | 49 | M | M250IT@16 | 2c |
|  | Italy | 2016 | 76 | F | M251IT@16 | 2c |
|  | Italy | 2016 | 82 | F | M252IT@16 | 2c |
|  | Italy | 2016 | 89 | F | M253IT@16 | 2c |
|  | Italy | 2016 | 78 | F | M254IT@16 | 2c |
|  | Italy | 2016 | 84 | F | M255IT@16 | 2c |
|  | Italy | 2016 | 68 | M | M256IT@16 | 2c |
|  | Italy | 2016 | 79 | F | M257IT@16 | 2c |
|  | Italy | 2016 | 72 | M | M258IT@16 | 2c |
|  | Italy | 2016 | 74 | F | M259IT@16 | 2c |
|  | Italy | 2016 | 54 | F | M260IT@16 | 2c |
|  | Italy | 2016 | 77 | M | M261IT@16 | 2c |
|  | Italy | 2016 | 70 | F | M262IT@16 | 2c |
|  | Italy | 2011 | 77 | M | M263IT@11 | 2c |
|  | Italy | 2011 |  | F | M264IT@11 | 2c |
|  | Italy | 2011 | 79 | F | M265IT@11 | 2c |
|  | Italy | 2011 | 81 | M | M266IT@11 | 2c |
|  | Italy | 2011 | 74 | F | M267IT@11 | 2c |
|  | Italy | 2011 | 87 | M | M268IT@11 | 2c |
|  | Italy | 2012 | 57 | F | M269IT@12 | 2c |
|  | Italy | 2012 | 59 | F | M270IT@12 | 2c |
|  | Italy | 2012 | 70 | M | M271IT@12 | 2c |
|  | Italy | 2012 | 77 | F | M272IT@12 | 2c |
|  | Italy | 2012 | 17 | F | M273IT@12 | 2c |
|  | Italy | 2012 | 53 | M | M274IT@12 | 2c |
|  | Italy | 2012 | 58 | F | M275IT@12 | 2c |
|  | Italy | 2012 | 53 | M | M276IT@12 | 2c |
|  | Italy | 2012 | 76 | F | M277IT@12 | 2c |
|  | Italy | 2012 | 90 | M | M278IT@12 | 2c |
|  | Italy | 2012 | 49 | F | M279IT@12 | 2c |
|  | Italy | 2012 | 89 | F | M280IT@12 | 2c |
|  | Italy | 2012 | 74 | F | M281IT@12 | 2c |
|  | Italy | 2012 | 76 | F | M282IT@12 | 2c |
|  | Italy | 2012 | 82 | F | M283IT@12 | 2c |
|  | Italy | 2012 | 67 | F | M284IT@12 | 2c |
|  | Italy | 2012 | 84 | M | M285IT@12 | 2c |
|  | Italy | 2012 | 41 | F | M286IT@12 | 2c |
|  | Italy | 2013 | 51 | M | M287IT@13 | 2c |
|  | Italy | 2013 | 76 | M | M289IT@13 | 2c |
|  | Italy | 2013 | 81 | F | M290IT@13 | 2c |
|  | Italy | 2013 | 57 | F | M291IT@13 | 2c |
|  | Italy | 2013 | 71 | M | M292IT@13 | 2c |
|  | Italy | 2013 | 80 | M | M293IT@13 | 2c |
|  | Italy | 2013 | 72 | F | M294IT@13 | 2c |
|  | Italy | 2013 | 56 | M | M295IT@13 | 2c |
|  | Italy | 2013 | 68 | M | M296IT@13 | 2c |
|  | Italy | 2013 | 32 | M | M297IT@13 | 2c |
|  | Italy | 2013 | 84 | M | M299IT@13 | 2c |
|  | Italy | 2013 | 66 | F | M300IT@13 | 2c |
|  | Italy | 2013 | 56 | M | M301IT@13 | 2c |
|  | Italy | 2013 | 54 | F | M302IT@13 | 2c |
|  | Italy | 2013 | 42 | M | M303IT@13 | 2c |
|  | Italy | 2013 | 51 | M | M304IT@13 | 2c |
|  | Italy | 2013 | 73 | M | M305IT@13 | 2c |
|  | Italy | 2013 | 73 | M | M306IT@13 | 2c |
|  | Italy | 2013 | 81 | F | M307IT@13 | 2c |
|  | Italy | 2013 | 77 | F | M308IT@13 | 2c |
|  | Italy | 2013 | 76 | F | M309IT@13 | 2c |
|  | Italy | 2014 | 74 | F | M311IT@14 | 2c |
|  | Italy | 2014 | 72 | F | M312IT@14 | 2c |
|  | Italy | 2014 | 85 | F | M313IT@14 | 2c |
|  | Italy | 2014 | 63 | F | M314IT@14 | 2c |
|  | Italy | 2014 | 29 | M | M315IT@14 | 2b |
|  | Italy | 2014 | 60 | F | M316IT@14 | 2c |
|  | Italy | 2014 | 81 | F | M317IT@14 | 2b |
|  | Italy | 2014 | 80 | F | M318IT@14 | 2c |
|  | Italy | 2014 | 63 | F | M319IT@14 | 2c |
|  | Italy | 2014 | 76 | F | M320IT@14 | 2c |
|  | Italy | 2014 | 57 | M | M321IT@14 | 2b |
|  | Italy | 2014 | 88 | F | M322IT@14 | 2c |
|  | Italy | 2014 | 72 | F | M323IT@14 | 2c |
|  | Italy | 2014 | 11 | F | M324IT@14 | 2c |
|  | Italy | 2014 | 39 | F | M325IT@14 | 2c |
|  | Italy | 2014 | 70 | M | M326IT@14 | 2c |
|  | Italy | 2014 | 69 | M | M327IT@14 | 2c |
|  | Italy | 2014 | 84 | M | M328IT@14 | 2c |
|  | Italy | 2014 | 60 | M | M329IT@14 | 2b |
|  | Italy | 2014 | 38 | F | M330IT@14 | 2c |
|  | Italy | 2014 | 61 | F | M331IT@14 | 2c |
|  | Italy | 2014 | 87 | F | M332IT@14 | 2c |
|  | Italy | 2014 | 80 | F | M333IT@14 | 2c |
|  | Italy | 2014 | 74 | F | M334IT@14 | 2c |
|  | Italy | 2014 | 75 | F | M335IT@14 | 2c |
|  | Italy | 2015 | 72 | M | M336IT@15 | 2c |
|  | Italy | 2015 | 87 | M | M337IT@15 | 2c |
|  | Italy | 2015 | 54 | M | M338IT@15 | 2c |
|  | Italy | 2015 | 68 | F | M339IT@15 | 2c |
|  | Italy | 2015 | 80 | F | R340IT@15 | 2c |
|  | Italy | 2013 | 80 | M | R341IT@13 | 2c |
|  | Italy | 2016 | 80 | F | R342IT@16 | 2c |
|  | Italy | 2013 | 73 | M | R343IT@13 | 2c |
|  | Italy | 2014 | 73 | F | R344IT@14 | 2c |
|  | Italy | 2013 | 84 | F | R345IT@13 | 2c |
|  | Italy | 2015 | 78 | M | R346IT@15 | 2c |
|  | Italy | 2013 | 62 | F | R347IT@13 | 2c |
|  | Italy | 2016 | 74 | F | R348IT@16 | 2c |
|  | Italy | 2016 | 83 | F | R350IT@16 | 2c |
|  | Italy | 2014 | 56 | M | R351IT@14 | 2c |
|  | Italy | 2014 | 83 | F | R352IT@14 | 2c |
|  | Italy | 2016 | 93 | M | R353IT@16 | 2c |
|  | Italy | 2015 | 76 | F | R354IT@15 | 2c |
|  | Italy | 2015 | 63 | F | R355IT@15 | 2c |
|  | Italy | 2015 | 64 | M | R356IT@15 | 2c |
|  | Italy | 2013 | 77 | M | R357IT@13 | 2c |
|  | Italy | 2015 | 62 | M | R358IT@15 | 2c |
|  | Italy | 2014 | 76 | M | R359IT@14 | 2c |
|  | Italy | 2015 | 88 | M | R360IT@15 | 2c |
|  | Italy | 2016 | 60 | F | R361IT@16 | 2c |
|  | Italy | 2013 | 76 | F | R362IT@13 | 2c |
|  | Italy | 2015 | 68 | M | R363IT@15 | 2c |
|  | Italy | 2015 | 76 | F | R364IT@15 | 2c |
|  | Italy | 2014 | 53 | F | R365IT@14 | 2c |
|  | Italy | 2011 | 69 | F | R366IT@11 | 2c |
|  | Italy | 2016 | 51 | M | S367IT@16 | 2c |
|  | Italy | 2015 | 81 | F | S368IT@15 | 2c |
|  | Italy | 2015 | 80 | F | S369IT@15 | 2c |
|  | Italy | 2016 | 61 | F | S370IT@16 | 2c |
|  | Italy | 2015 | 76 | F | S371IT@15 | 2c |
|  | Italy | 2016 | 52 | F | S372IT@16 | 2c |
|  | Italy | 2015 | 77 | F | S373IT@15 | 2c |
|  | Italy | 2016 | 77 | M | S374IT@16 | 2c |
|  | Italy | 2016 | 40 | F | S375IT@16 | 2c |
|  | Italy | 2016 | 77 | F | S376IT@16 | 2c |
|  | Italy | 2015 | 80 | F | S377IT@15 | 2c |
|  | Italy | 2016 | 73 | F | S378IT@16 | 2c |
|  | Italy | 2010 |  |  | I379IT@10 | 2c |
|  | Italy | 2010 |  |  | I380IT@10 | 2c |
|  | Italy | 2010 |  |  | I381IT@10 | 2c |
|  | Italy | 2010 |  |  | I382IT@10 | 2c |
|  | Italy | 2010 |  |  | I383IT@10 | 2c |
|  | Italy | 2010 |  |  | I384IT@10 | 2c |
|  | Italy | 2010 |  |  | I385IT@10 | 2c |
|  | Italy | 2010 |  |  | I386IT@10 | 2c |
|  | Italy | 2010 |  |  | I387IT@10 | 2c |
|  | Italy | 2010 |  |  | I388IT@10 | 2c |
|  | Italy | 2010 |  |  | I389IT@10 | 2c |
|  | Italy | 2010 |  |  | I390IT@10 | 2c |
|  | Italy | 2010 |  |  | I391IT@10 | 2c |
|  | Italy | 2010 |  |  | I392IT@10 | 2c |
|  | Italy | 2010 |  |  | I393IT@10 | 2c |
|  | Italy | 2010 |  |  | I394IT@10 | 2c |
|  | Italy | 2011 |  |  | I395IT@11 | 2c |
|  | Italy | 2008 |  |  | I396IT@08 | 2c |
|  | Italy | 2011 |  |  | I397IT@11 | 2c |
|  | Italy | 2011 |  |  | I398IT@11 | 2c |
|  | Italy | 2007 |  |  | I399IT@07 | 2c |
|  | Italy | 2011 |  |  | I400IT@11 | 2c |
|  | Italy | 2011 |  |  | I401IT@11 | 2c |
|  | Italy | 2011 |  |  | I402IT@11 | 2c |
|  | Italy | 2008 |  |  | I403IT@08 | 2c |
|  | Italy | 2008 |  |  | I404IT@08 | 2c |
|  | Italy | 2010 |  |  | I405IT@10 | 2c |
|  | Italy | 2009 |  |  | I406IT@09 | 2c |
|  | Italy | 2007 |  |  | I407IT@07 | 2c |
|  | Italy | 2007 |  |  | I408IT@07 | 2c |
|  | Italy | 2007 |  |  | I409IT@07 | 2c |
|  | Italy | 2007 |  |  | I410IT@07 | 2c |
|  | Italy | 2007 |  |  | I411IT@07 | 2c |
|  | Italy | 2008 |  |  | I412IT@08 | 2c |
|  | Italy | 2007 |  |  | I413IT@07 | 2c |
|  | Italy | 2008 |  |  | I414IT@08 | 2c |
|  | Italy | 2008 |  |  | I415IT@08 | 2c |
|  | Italy | 2009 |  |  | I416IT@09 | 2c |
|  | Italy | 2009 |  |  | I417IT@09 | 2c |
|  | Italy | 2009 |  |  | I418IT@09 | 2c |
|  | Italy | 2010 |  |  | I419IT@10 | 2c |
|  | Italy | 2010 |  |  | I420IT@10 | 2c |
|  | Italy | 2010 |  |  | I421IT@10 | 2c |
|  | Italy | 2009 |  |  | I422IT@09 | 2c |
|  | Italy | 2007 |  |  | I423IT@07 | 2c |
|  | Italy | 2007 |  |  | I424IT@07 | 2c |
|  | Italy | 2007 |  |  | I425IT@07 | 2c |
|  | Italy | 2007 |  |  | I426IT@07 | 2c |
|  | Italy | 2007 |  |  | I427IT@07 | 2c |
|  | Italy | 2007 |  |  | I428IT@07 | 2c |
|  | Italy | 2008 |  |  | I429IT@08 | 2c |
|  | Italy | 2001 |  |  | I430IT@01 | 2c |
|  | Italy | 2011 |  |  | I431IT@11 | 2c |
|  | Italy | 2008 |  |  | I432IT@08 | 2c |
|  | Italy | 2009 |  |  | I433IT@09 | 2c |
| D49761 | Indonesia | 2004 |  |  | 434ID@04 | 2e |
| D49777 | Indonesia | 1997 |  |  | 435ID@97 | 2f |
| JF722455 | Dominican Republic | 1998 |  |  | 436DO@98 | 2r |
| JF722457 | Holland | 1999 |  |  | 437NL@99 | 2b |
| JF722458 | Holland | 2002 |  |  | 438NL@02 | 2b |
| JF722459 | Holland | 1997 |  |  | 439NL@97 | 2b |
| JF722460 | Holland | 2000 |  |  | 440NL@00 | 2b |
| JF722461 | Indonesia | 2001 |  |  | 441ID@01 | 2e |
| JF722463 | Holland | 1990 |  |  | 442NL@90 | 2b |
| JF722464 | Holland | 1986 |  |  | 443NL@86 | 2b |
| JF722467 | Holland | 1992 |  |  | 444NL@92 | 2b |
| JF722468 | Holland | 1994 |  |  | 445NL@94 | 2b |
| JF722469 | Holland | 1985 |  |  | 446NL@85 | 2b |
| JF722470 | Holland | 1990 |  |  | 447NL@90 | 2b |
| JF722472 | Holland | 2008 |  |  | 448NL@08 | 2b |
| JF722473 | Holland | 2008 |  |  | 449NL@08 | 2b |
| JF722474 | Holland | 2006 |  |  | 450NL@06 | 2b |
| JF722481 | Holland | 2004 |  |  | 451NL@04 | 2b |
| JF722482 | Holland | 2004 |  |  | 452NL@04 | 2b |
| JF722483 | Holland | 2004 |  |  | 453NL@04 | 2b |
| JF722485 | Holland | 2007 |  |  | 454NL@07 | 2b |
| JF722486 | Holland | 2004 |  |  | 455NL@04 | 2b |
| JF722488 | Holland | 2005 |  |  | 456NL@05 | 2b |
| JF722489 | Holland | 2005 |  |  | 457NL@05 | 2b |
| JF722490 | Holland | 2005 |  |  | 458NL@05 | 2b |
| JF722491 | Holland | 2006 |  |  | 459NL@06 | 2b |
| JF722492 | Holland | 2006 |  |  | 460NL@06 | 2b |
| JF722493 | Holland | 2004 |  |  | 461NL@04 | 2b |
| JF722494 | Holland | 2006 |  |  | 462NL@06 | 2b |
| JF722495 | Holland | 2004 |  |  | 463NL@04 | 2b |
| JF722496 | Holland | 2001 |  |  | 464NL@01 | 2b |
| JF722497 | Holland | 2004 |  |  | 465NL@04 | 2b |
| JF722498 | Holland | 2007 |  |  | 466NL@07 | 2b |
| JF722499 | Holland | 2005 |  |  | 467NL@05 | 2b |
| JF722501 | Holland | 2006 |  |  | 468NL@06 | 2b |
| JF722502 | Holland | 1988 |  |  | 469NL@88 | 2b |
| JF722503 | Holland | 2005 |  |  | 470NL@05 | 2b |
| JF722504 | Holland | 2007 |  |  | 471NL@07 | 2b |
| JF722505 | Holland | 2008 |  |  | 472NL@08 | 2b |
| JF722510 | Holland | 2005 |  |  | 473NL@05 | 2b |
| JF722512 | Holland | 2001 |  |  | 474NL@01 | 2b |
| JF722513 | Holland | 2002 |  |  | 475NL@02 | 2b |
| JF722515 | Morocco | 2005 |  |  | 476MA@05 | 2i |
| JF722516 | Holland | 2003 |  |  | 477NL@03 | 2b |
| JF722518 | Morocco | 2006 |  |  | 478MA@06 | 2i |
| JF722519 | Holland | 2004 |  |  | 479NL@04 | 2b |
| JF722520 | Holland | 2004 |  |  | 480NL@04 | 2b |
| JF722522 | Algeria | 2001 |  |  | 481DZ@01 | 2i |
| JF722523 | Holland | 2002 |  |  | 482NL@02 | 2b |
| JF722524 | Morocco | 2003 |  |  | 483MA@03 | 2i |
| JF722525 | Indonesia | 2000 |  |  | 484ID@00 | 2e |
| JF722526 | Holland | 2003 |  |  | 485NL@03 | 2b |
| JF722527 | Holland | 2007 |  |  | 486NL@07 | 2b |
| JF722529 | Morocco | 2002 |  |  | 487MA@02 | 2i |
| JF722532 | Indonesia | 2001 |  |  | 488ID@01 | 2b |
| JF722534 | Holland | 2002 |  |  | 489NL@02 | 2b |
| JF722535 | Morocco | 2000 |  |  | 490MA@00 | 2i |
| JF722536 | Suriname | 2001 |  |  | 491SR@01 | 2f |
| JF722537 | Suriname | 2000 |  |  | 492SR@00 | 2e |
| JF722538 | Holland | 2004 |  |  | 493NL@04 | 2b |
| JF722541 | Holland | 2001 |  |  | 494NL@01 | 2b |
| JF722542 | Holland | 2000 |  |  | 495NL@00 | 2j |
| JF722546 | Holland | 2002 |  |  | 496NL@02 | 2b |
| JF722547 | Morocco | 2004 |  |  | 497MA@04 | 2i |
| JF722548 | Holland | 2002 |  |  | 498NL@02 | 2b |
| JF722549 | Holland | 2003 |  |  | 499NL@03 | 2b |
| JF722553 | Morocco | 2004 |  |  | 500MA@04 | 2i |
| JF722554 | Suriname | 2002 |  |  | 501SR@02 | 2e |
| JF722555 | Holland | 2002 |  |  | 502NL@02 | 2e |
| JF722557 | Holland | 2002 |  |  | 503NL@02 | 2b |
| JF722559 | Morocco | 2005 |  |  | 504MA@05 | 2i |
| JF722565 | Holland | 2003 |  |  | 505NL@03 | 2b |
| JF722570 | Turkey | 2001 |  |  | 506NL@01 | 2b |
| JF722571 | Suriname | 2000 |  |  | 507SR@00 | 2f |
| JF722572 | Suriname | 2006 |  |  | 508SR@06 | 2e |
| JF722574 | Holland | 2003 |  |  | 509NL@03 | 2b |
| JF722575 | Holland | 2000 |  |  | 510NL@00 | 2b |
| JF722577 | Holland | 2003 |  |  | 511NL@03 | 2b |
| JF722579 | Holland | 2002 |  |  | 512NL@02 | 2b |
| JF722582 | Holland | 2008 |  |  | 513NL@08 | 2b |
| JF722586 | Suriname | 2004 |  |  | 514SR@04 | 2f |
| JF722590 | Suriname | 2006 |  |  | 515SR@06 | 2f |
| JF722591 | Holland | 2005 |  |  | 516NL@05 | 2b |
| JF722594 | Holland | 2004 |  |  | 517NL@04 | 2b |
| JF722596 | Suriname | 2001 |  |  | 518SR@01 | 2f |
| JF722607 | Holland | 2002 |  |  | 519NL@02 | 2b |
| JF722608 | Holland | 2008 |  |  | 520NL@08 | 2b |
| JF722611 | Holland | 2008 |  |  | 521NL@08 | 2b |
| JF722614 | Holland | 2006 |  |  | 522NL@06 | 2b |
| JF722615 | Holland | 2004 |  |  | 523NL@04 | 2b |
| JF722616 | Holland | 2008 |  |  | 524NL@08 | 2b |
| D50409 | Italy | 1994 |  |  | 527IT@94 | 2c |
