# Supplementary figures and images for: Time and Mode of Epidemic HCV-2 Subtypes Spreading in Europe: Phylodynamics in Italy and Albania
Source: Diagnostics (Basel). 2021 Feb 17;11(2):327. doi: 10.3390/diagnostics11020327 (PMC7922790; doi:10.3390/diagnostics11020327)

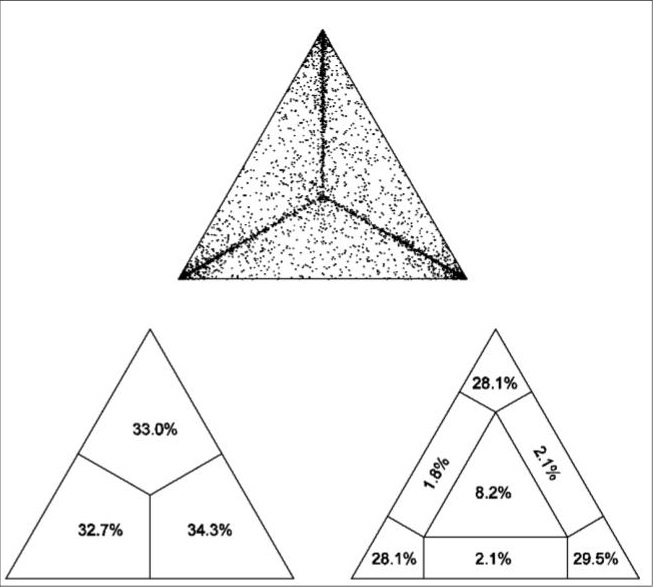

Supplement: Supplementary file 1 [file diagnostics-11-00327-s001.zip › Supplementary files/Supplementary figure 1_300dpi.tif]
